# Preconditioning before and during oxygenators use: “The Road to Extinction of High-pressure Excursion”

**DOI:** 10.1051/ject/2025009

**Published:** 2025-06-16

**Authors:** Ignazio Condello

**Affiliations:** 1 Department of Cardiac Surgery, Anthea Hospital, GVM Care & Research Via Camillo Rosalba 35/37 70124 Bari Italy

**Figure 1:**
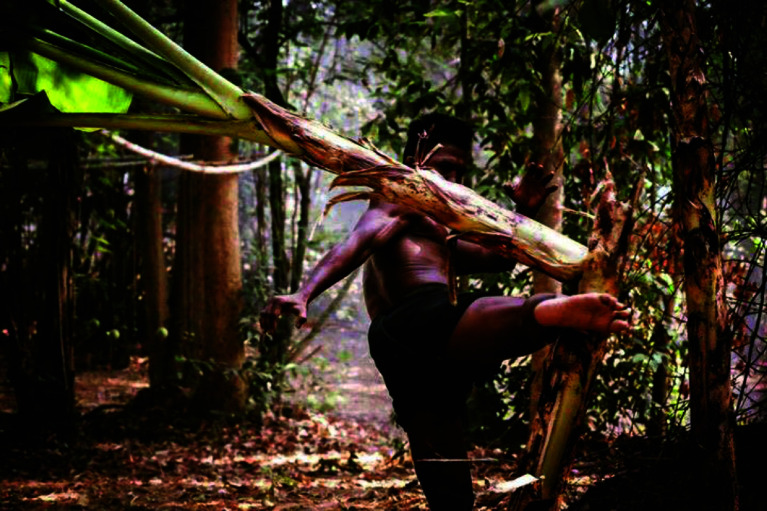
Preconditioning during Muay Thai training.

Preconditioning, in any context, refers to preparing for future stresses through gradual, controlled exposure to specific stimuli. This concept is vividly embodied in the training routines of Muay Thai fighters. These athletes condition themselves to pain by repeatedly striking their shins against hard objects, such as banana trees, effectively desensitizing their bones and tissues to the shocks they will endure in combat (Figure 1) [1]. Similarly, in the medical field, particularly in the setting of cardiopulmonary bypass (CPB), we apply the principle of preconditioning to enhance the resilience of biological systems specifically, oxygenators against potential traumatic events such as high-pressure excursions (HPE). HPE in oxygenators during CPB represents a significant and potentially traumatic event for both the patient and the surgical team. Characterized by a rapid increase in oxygenator inlet pressure, HPE can drastically limit blood flow, thereby jeopardizing the efficacy of CPB and patient safety. We read with great interest the article “High pressure excursion in a radial design oxygenator” by Ashley Svec et al. This insightful exploration of HPE during CPB and the subsequent management strategies employed contributes significantly to our understanding of the complexities associated with managing HPE in CPB [2]. In our experience on normothermic procedures the preconditioning techniques, such as Retrograde Autologous Priming (RAP), play a crucial role in mitigating these risks by preparing the oxygenator system in a way that enhances its resilience to sudden pressure changes. These techniques not only improve the stability of the system but also help to prevent the occurrence of such traumatic events during surgery. In cases where patients exhibit high hematocrit, the priming solution is mixed with the patient’s blood to ensure a gradual introduction of blood into the oxygenator, moderating the shock to the system and minimizing the risk of HPE. Conversely, for patients with low hematocrit, the priming solution is completely replaced with blood, to improve oxygen delivery during the bypass. Once the blood has been appropriately mixed with the priming solution or replaced entirely, the next critical step involves circulating this mixture through the shunt or recirculation loop. During this phase, it is crucial to set the temperature to normothermia, specifically maintaining it at 36 °Celsius. This temperature setting helps in stabilizing the patient’s physiological condition and ensures that the blood properties are optimal for CPB, reducing the potential for thermal shock and other related complications [3]. In hypothermic procedures the context of radial oxygenator design, utilizing cell saver technology to perform platelet apheresis before the institution of hypothermia management could potentially mitigate the risks associated with thermal excursions during hypothermic phases of CPB and preserve platelet functionality and count. Platelet apheresis using a cell saver is a specialized medical procedure designed to selectively separate and collect platelets from a patient’s blood, while the other blood components, such as red blood cells and plasma, are either returned to the patient or further managed as needed. This approach might reduce the incidence of HPE, enhancing overall patient safety and the effectiveness of the procedure [4]. Despite these advanced techniques, including RAP, it is noteworthy that in the article presented by our colleagues, an HPE still occurred at a venous blood return temperature of 31.4 °C during the cooling phase. At the end of the procedure after the various replacements of the oxygenator models, a platelet count of 17,000/mL was found. This instance underscores the multifactorial nature of HPE and highlights the need for comprehensive strategies that integrate both mechanical and biological approaches to effectively manage the complexities of CPB in normothermic and hypothermic management. Continued exploration and refinement of these techniques are crucial. By potentially adjusting the extent of platelet apheresis based on real-time assessments of platelet function and quantity, which can be facilitated by the cell saver’s capabilities, we could further enhance the procedural outcomes. I would be interested to hear if your team or others in the field have utilized the platelet apheresis function of cell savers in conjunction with RAP, and what the outcomes of such integrated approaches have been. Specifically, have these practices influenced the incidence of bleeding, the need for transfusion, or the occurrence of HPE during CPB? By sharing our experiences and querying the experiences of others, we aim to encourage a deeper understanding and wider adoption of these comprehensive care strategies, which could lead to improved standards of care in CPB procedures and better patient outcomes. Finally, it is crucial to address how consistently the pressure before the oxygenator is monitored, as understanding this practice may be pivotal in preventing high-pressure excursions. This question should form a central part of our ongoing dialogue in refining CPB protocols. Thank you again for the opportunity to contribute to this important conversation. I look forward to your insights and further advancements in the field of extracorporeal technology.
